# Age‐Specific Gonadotoxic Alkylating‐Agent Exposure in Pediatric and Reproductive‐Age Females: A Claims‐Based Study Using Cyclophosphamide Equivalent Dose

**DOI:** 10.1002/rmb2.70084

**Published:** 2026-08-02

**Authors:** Kentaro Taniguchi, Mizuki Ohashi, Yuji Tanaka, Tetsuro Hanada, Akie Takebayashi, Akimasa Takahashi, Tsukuru Amano, Shunichiro Tsuji

**Affiliations:** ^1^ Department of Obstetrics and Gynaecology Shiga University of Medical Science Otsu Shiga Japan

**Keywords:** administrative claims database, alkylating agents, cyclophosphamide equivalent dose, fertility preservation, gonadotoxicity

## Abstract

**Purpose:**

To describe age‐specific patterns of gonadotoxic alkylating‐agent exposure and evaluate how the cyclophosphamide equivalent dose (CED) framework captures real‐world exposure relevant to fertility preservation in females aged 0–43 years.

**Methods:**

This descriptive study used a Japanese claims database. Patients were classified as pediatric (< 15 years) or reproductive‐age (15–43 years). Twelve alkylating agents were evaluated, and six CED‐calculable agents were converted to CED.

**Results:**

Among approximately 3.86 million females, 0.04% of the pediatric cohort and 0.16% of the reproductive‐age cohort were exposed to at least one alkylating agent. Total CED identified at least intermediate‐risk exposure in 33% of exposed pediatric and 14% of exposed reproductive‐age patients. Compared with single‐agent assessment, summed total CED identified additional patients mainly in the pediatric group, corresponding to relative increases of 20.2% and 0.3%, respectively. Agents not included in the current CED formula accounted for 8.7% of all alkylating‐agent prescription records, approximately 90% of which were observed in reproductive‐age patients.

**Conclusions:**

CED captured alkylating‐agent exposure differently by age group: summed CED added exposure information mainly in pediatric patients, whereas agents lacking conversion coefficients limited exposure capture in reproductive‐age females. These findings support refinement of CED‐based assessment while emphasizing exposure capture, not outcome prediction.

## 1 Introduction

Alkylating agents are widely used in anticancer and immunosuppressive therapies for both malignant and non‐malignant diseases in female patients. Beyond oncology, fertility preservation has emerged as a critical concern for women receiving gonadotoxic therapy for systemic autoimmune diseases [1]. These treatments involve the most gonadotoxic systemic agents used in clinical practice [2]. Consequently, assessment of alkylating‐agent exposure before treatment is important to identify patients who may benefit from timely counseling and appropriate cryopreservation strategies [3].

As ovarian toxicity generally increases with cumulative exposure, accurate description of the total alkylating‐agent burden is important for fertility‐related counseling. However, alkylating agents constitute a heterogeneous group of drugs with varying gonadotoxic potentials and dosing patterns, and can be used in combination. The cyclophosphamide equivalent dose (CED) was developed to standardize exposure across drugs with different potencies [4] and has since been incorporated into gonadotoxicity risk assessment frameworks, particularly in pediatric and adolescent/young adult settings [5, 6]. The CED provides a common framework for describing cumulative alkylating‐agent exposure, and its clinical relevance to female gonadal outcomes has been supported by studies linking cumulative alkylating‐agent burden to premature ovarian insufficiency and fertility impairment [7, 8, 9].

Nevertheless, real‐world patterns of alkylating‐agent use vary significantly across diseases and age groups. The extent to which CED‐based integration of multi‐agent exposure adds information beyond single‐agent assessment in clinical practice remains poorly understood. Furthermore, whether the current CED framework adequately captures comprehensive real‐world exposure, particularly as several alkylating agents are not included in the existing formula, remains unclear.

To address these gaps, this study aimed to characterize age‐specific prescription patterns of alkylating agents across diseases, quantify cumulative multi‐agent dose exposure, and evaluate the extent to which the current CED framework captures alkylating‐agent exposure in females aged 0–43 years using data from a Japanese claims database. Our findings may improve understanding of real‐world alkylating‐agent exposure and help clarify the descriptive utility and limitations of the current CED framework in females across age groups.

## 2 Materials and Methods

### 2.1 Ethical Approval

This study was conducted in accordance with the Declaration of Helsinki and the Japanese Ethical Guidelines for Medical and Biological Research Involving Human Subjects. The requirement for informed consent was waived because of the use of de‐identified data. The study protocol was approved by the Institutional Review Board of the Shiga University of Medical Science (R2026‐007).

### 2.2 Data Source and Study Population

We conducted a descriptive analysis using the Japanese administrative claims database provided by the Japan Medical Data Centre (JMDC Inc., Tokyo, Japan). The JMDC database contains anonymized, time‐stamped claims data collected from employee‐based health insurance societies in Japan, which primarily encompasses employees of medium‐to‐large companies and their dependents. Since these insurance societies generally enroll individuals aged < 75 years, the database predominantly represents the working‐age population and their family members. Available variables include sex, year and month of birth, recorded diagnoses, medical procedures, and prescription records [10].

The study period spanned from 2005 to 2023. We identified females aged 0–43 years for whom claim records were available during the study period. Sex was determined based on the registration recorded in the database. Cohort entry was defined as the first observable month in the database during the study period, with the start of the observable follow‐up defined accordingly. To construct the analytical cohort, we excluded potential duplicate entries resulting from changes in insurance enrollment. Potential duplicates were defined as individuals with different identifiers but identical sex and birth year/month, and who exhibited overlapping or sequential enrollment patterns indicating re‐registration. Individuals with < 2 years of observable follow‐up after cohort entry were also excluded, and the final analytical cohort included the remaining individuals.

### 2.3 Study Drugs and Exposure Ascertainment

We examined 12 alkylating agents available in the database: cyclophosphamide, ifosfamide, procarbazine, melphalan, thiotepa, busulfan, nimustine, ranimustine, temozolomide, bendamustine, streptozocin, and dacarbazine. Drug exposure was determined using inpatient, outpatient, and prescription claims. Prescription frequency was defined as the number of unique patients with at least one record for a specific alkylating agent during the study period. For patient‐level exposure assessment, all recorded doses of each drug were summed from the start of the observable follow‐up until the day before the patient turned 44 to obtain the cumulative exposure. Any exposures accrued at age ≥ 44 years were excluded.

### 2.4 Age Stratification

Patients were classified into two primary age groups: pediatric (< 15 years) and reproductive age (15–43 years). An upper age limit of 43 years was selected to reflect the typical reproductive age range targeted for fertility preservation counseling in clinical practice.

### 2.5 Cumulative Dose Per Square Meter

Since individual body surface area (BSA) was not consistently available in the claims data, the cumulative dose per square meter was estimated using age‐specific standard BSA values. These standardized values were derived from the age‐specific mean height and weight reported in table 14 of the 2023 National Health and Nutrition Survey, Japan (e‐Stat) [11], calculated via the Mosteller formula [12]. Given that the survey provides data only for individuals aged ≥ 1 year, a value for age 0 was unavailable; therefore, a BSA of 0.44 m^2^ was assigned based on the mean BSA at age 1 (0.445 m^2^). Age at exposure was defined as the patient's age on the date of each prescription claim. For each alkylating agent record, the recorded dose was divided by the standard BSA corresponding to the patient's age at exposure, and these resulting values were then summed per patient to obtain the total cumulative exposure in mg/m^2^.

### 2.6 CED

The CED was calculated using the method described by Green et al. [4] and is defined as follows:

$$\begin{array}{c} \mathrm{CED}\;\left(\mathrm{mg}/m^{2}\right)=1.0\times \text{cumulative cyclophosphamide dose}\;\left(\mathrm{mg}/m^{2}\right)\\ \;+0.244\times \text{cumulative ifosfamide dose}\;\left(\mathrm{mg}/m^{2}\right)+0.857\\ \;\times \text{cumulative procarbazine dose}\;\left(\mathrm{mg}/m^{2}\right)+14.286\\ \;\times \text{cumulative chlorambucil dose}\;\left(\mathrm{mg}/m^{2}\right)+15.0\\ \;\times \text{cumulative BCNU dose}\;\left(\mathrm{mg}/m^{2}\right)+16.0\\ \;\times \text{cumulative CCNU dose}\;\left(\mathrm{mg}/m^{2}\right)+40\\ \;\times \text{cumulative melphalan dose}\;\left(\mathrm{mg}/m^{2}\right)+50\\ \;\times \text{cumulative thiotepa dose}\;\left(\mathrm{mg}/m^{2}\right)+100\\ \;\times \text{cumulative mechlorethamine dose}\;\left(\mathrm{mg}/m^{2}\right)+8.823\\ \;\times \text{cumulative busulfan dose}\;\left(\mathrm{mg}/m^{2}\right). \end{array}$$

In the present study, the CED was calculated for the six alkylating agents captured in the database and included in the published conversion formula: cyclophosphamide, ifosfamide, procarbazine, melphalan, thiotepa, and busulfan. Although the original formula also includes chlorambucil, carmustine (BCNU), lomustine (CCNU), and mechlorethamine, these agents were not evaluated because they were not available in the claims database. For each patient, the cumulative dose of each evaluable agent (mg/m^2^) was multiplied by its corresponding conversion factor, and the resulting values were summed to obtain the total CED.

### 2.7 CED‐Based Exposure Risk Categorization

CED‐based risk categories were used to classify age‐specific exposure levels relevant to fertility preservation counseling. This categorization was primarily based on the Pediatric Initiative Network risk stratification system [6] and the Japanese Society of Clinical Oncology guidelines [5]. It was further informed by reports highlighting treatment‐related gonadotoxic risk and fertility preservation needs in pediatric, adolescent, and young adult populations [13, 14].

In pediatric patients (< 15 years), CED values of < 8000 mg/m^2^, 8000–12,000 mg/m^2^, and > 12,000 mg/m^2^ were classified as low‐, intermediate‐, and high‐risk exposure, respectively. In reproductive‐age patients (15–43 years), the corresponding thresholds were < 4000 mg/m^2^, 4000–8,000 mg/m^2^, and > 8000 mg/m^2^. For patients who received alkylating agents both before and after the age of 15 years, reproductive‐age risk thresholds were applied. For the primary descriptive analysis, intermediate‐ and high‐risk exposures were combined into an ‘at least intermediate‐risk’ category to identify patients with exposure levels potentially relevant to fertility preservation counseling. Risk classification was based on the CED calculated from the six evaluable agents recorded in the database. Additionally, we identified patients whose exposure reached the ‘at least intermediate‐risk’ threshold only through integration of multiple agents, rather than any single evaluable agent alone.

### 2.8 Study Outcomes

The study outcomes were: (i) prescription frequency of the 12 analyzed alkylating agents; (ii) distribution of cumulative prescribed doses prior to CED conversion for the six agents included in the formula; (iii) distribution of agent‐specific CEDs for these six agents; (iv) distribution of total CED per patient, stratified by age group (pediatric vs. reproductive‐age); (v) number of additional patients identified as having at least intermediate‐risk exposure when utilizing the total CED rather than single‐agent assessment; and (vi) distribution of cumulative prescribed doses for alkylating agents not included in the current CED formula.

### 2.9 Statistical Analysis

Categorical variables are summarized as counts and percentages. Continuous exposure variables are presented descriptively and visualized using histograms. The histograms illustrate the cumulative prescribed doses prior to CED conversion, agent‐specific CEDs for the six evaluable agents, total CED per patient, and cumulative prescribed doses for alkylating agents not included in the current CED formula. No formal hypothesis testing was performed.

## 3 Results

### 3.1 Study Cohort and the Prescribing Patterns of Alkylating Agents

Among the 1,101,594 pediatric and 2,758,952 reproductive‐age females in the analytical cohort, 453 (0.04%) and 4439 (0.16%), respectively, received at least one alkylating agent during the study period, yielding 4892 exposed patients in total. The median follow‐up duration across these exposed patients was 63 months (interquartile range [IQR], 40–88 months).

**FIGURE 1:**
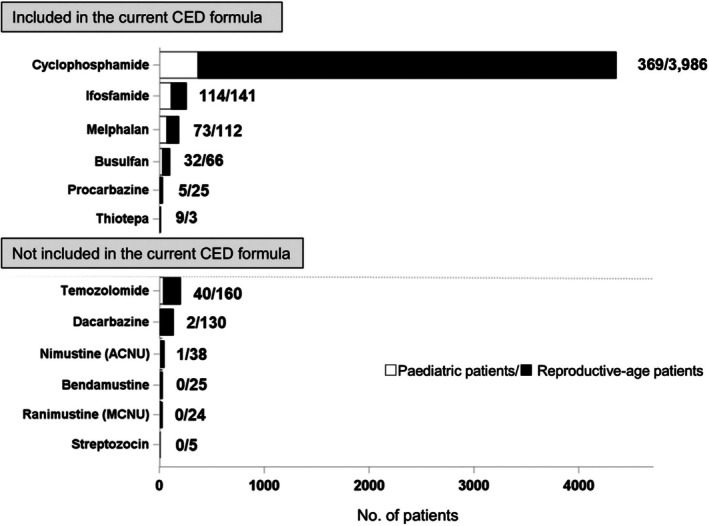
Distribution of alkylating‐agent exposure among females aged 0–43 years. Number of patients who received prescriptions for 12 alkylating agents during the study period (2005–2023), grouped according to whether each agent was included in the current cyclophosphamide equivalent dose (CED) formula. Each bar is divided by age group into pediatric (< 15 years; open bars) and reproductive‐age (15–43 years; solid bars) patients. The numbers adjacent to the bars indicate the number of pediatric and reproductive‐age patients treated with each agent. As individual patients may receive more than one alkylating agent, these counts across drugs are not mutually exclusive.

Figure 1 presents the prescription frequency of individual alkylating agents, categorized by their inclusion in the current CED formula. Cyclophosphamide was the most frequently prescribed alkylating agent in both pediatric and reproductive‐age patients, accounting for 81.5% and 89.8% of exposed patients, respectively. Other evaluable agents included in the current CED formula, such as ifosfamide, melphalan, busulfan, procarbazine, and thiotepa, were prescribed in both age groups. In contrast, alkylating agents not included in the current CED formula, such as dacarbazine, nimustine, ranimustine, bendamustine, streptozocin, and temozolomide, were prescribed predominantly to reproductive‐age patients, with minimal or no pediatric exposure observed for several agents. These findings indicate that real‐world alkylating‐agent exposure comprises both CED‐calculable and non‐CED domains with distinct age‐related prescription patterns.

### 3.2 CED‐Based Exposure Burden and Age‐Specific Distribution of Total CED

**FIGURE 2:**
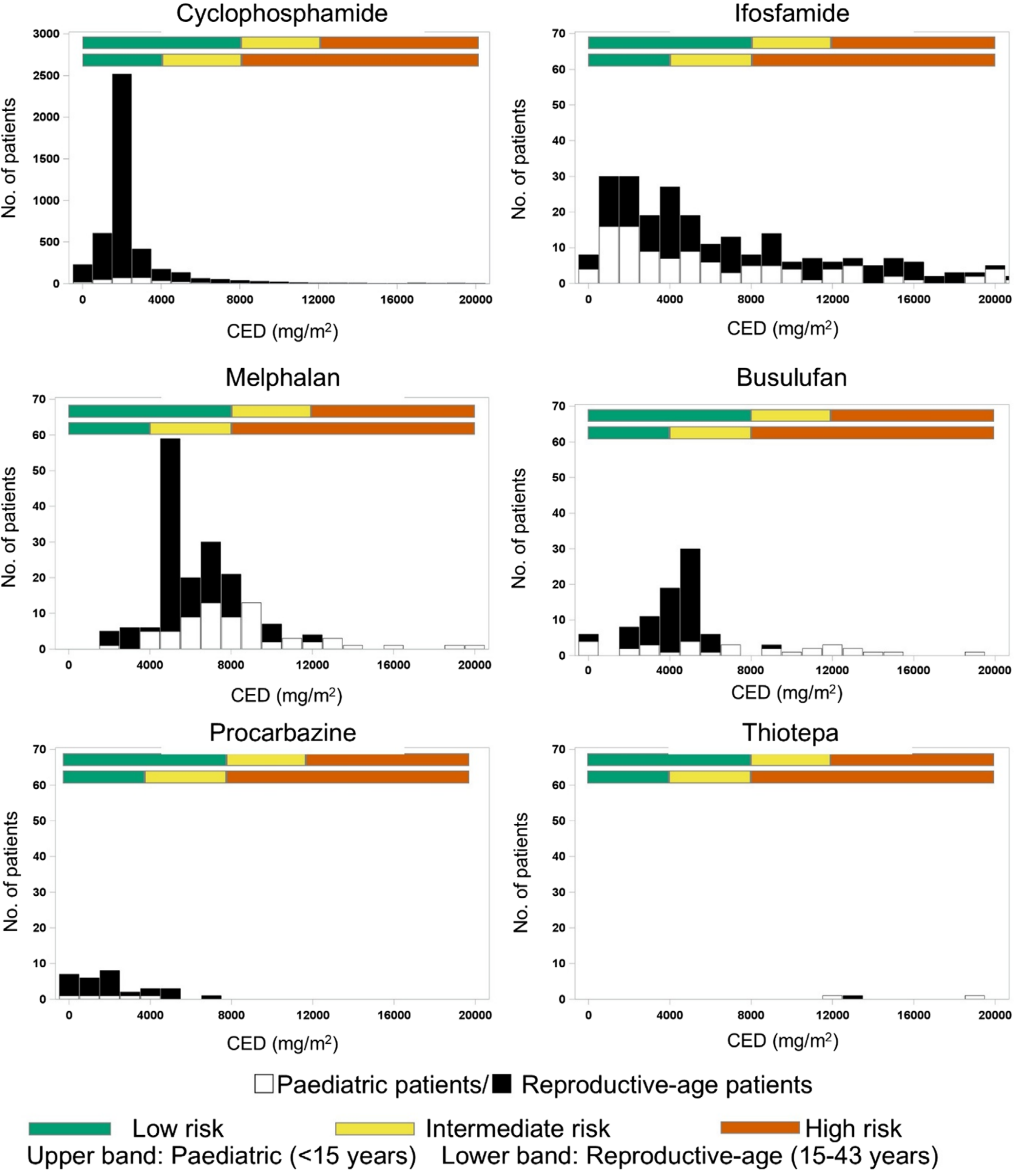
Distribution of agent‐specific cyclophosphamide equivalent dose (CED) for six evaluable alkylating agents. Distribution of cumulative exposure for the six alkylating agents converted to a cyclophosphamide equivalent dose (CED; mg/m^2^). Patients are stratified by age group as follows: Pediatric patients (< 15 years; open bars) and reproductive‐age patients (15–43 years; solid bars). The two horizontal bands at the top of each panel indicate the age‐specific CED‐based exposure categories applied to each cohort. The upper and lower bands correspond to pediatric and reproductive‐age patients, respectively. Green, yellow, and orange indicate low‐, intermediate‐, and high‐risk exposure, respectively. The x‐ and y‐axis scales differ across panels to accommodate between‐drug differences in the cumulative prescribed doses and prescription frequencies, particularly for cyclophosphamide.

Histograms of the cumulative prescribed doses prior to CED conversion for the six CED‐calculable agents are shown in Figure S1, and the corresponding agent‐specific exposure distributions following conversion are presented in Figure 2. After standardization by age‐specific body surface area and the application of agent‐specific conversion coefficients, the exposure distributions differed across agents. Single‐agent CED assessments showed marked differences in the frequency with which specific agents reached at least intermediate‐risk exposure. Cyclophosphamide, although the most frequently prescribed agent in both age groups, reached at least intermediate‐risk exposure in only 16.3% of pediatric and 10.9% of reproductive‐age patients. In contrast, the corresponding frequencies were higher for melphalan (54.8% vs. 91.1%), ifosfamide (38.6% vs. 70.2%), and busulfan (43.8% vs. 75.8%). Notably, the CED‐based evaluations revealed differences in exposure burden not apparent from prescription frequency alone.

**FIGURE 3:**
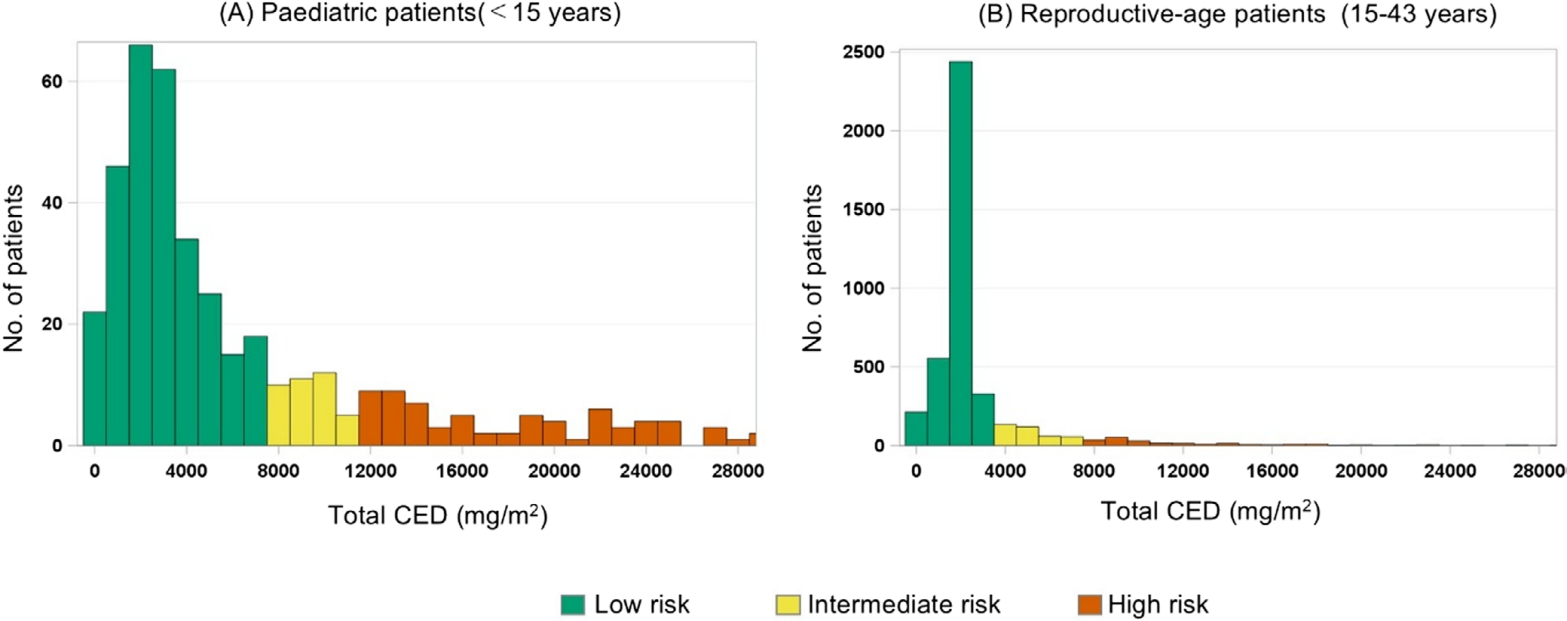
Distribution of total cyclophosphamide equivalent dose (CED) by age group. Distribution of the total cyclophosphamide equivalent dose (CED; mg/m^2^) per patient, calculated by summing exposures across evaluable alkylating agents. (A) Pediatric patients (< 15 years). (B) Reproductive‐age patients (15–43 years). Histogram bars are color‐coded according to age‐specific CED‐based exposure categories: Green indicates low‐risk exposure, yellow indicates intermediate‐risk exposure, and orange indicates high‐risk exposure. The x‐ and y‐axis scales differ across panels to accommodate variations in standard dosing regimens and prescription frequencies. Patients exposed both before and after the age of 15 years (*n* = 20) were assigned the reproductive‐age thresholds for the primary numerical classification but were excluded from the age‐stratified panels in Figure 3 to ensure distinct categorical presentation.

Figure 3 shows the distribution of the total CED at the patient level, calculated by integrating exposure across all evaluable alkylating agents. Among pediatric patients with a calculable total CED from single or multiple evaluable alkylating agents, the values were broadly distributed across the low‐, intermediate‐, and high‐risk ranges, with 33% of patients classified as having at least intermediate‐risk exposure. In contrast, in reproductive‐age patients, the total CED was relatively concentrated in the low‐risk range, and only 14% were classified as having at least intermediate‐risk exposure. Overall, cumulative patient‐level CED‐based exposure was more broadly distributed toward higher‐risk ranges in pediatric patients than in reproductive‐age patients.

### 3.3 Incremental Value and Limitations of the Current CED Framework

Table 1 shows the additional patients identified as having at least intermediate‐risk exposure when CED was aggregated across multiple evaluable alkylating agents rather than assessed on a single‐agent basis. The incremental yield of the summed total CED was concentrated predominantly in younger patients. Compared with single‐agent CED thresholds, summed total CED identified 25 additional pediatric patients and two additional reproductive‐age patients, representing relative increases of 20.2% and 0.3%, respectively. These findings indicate that integrating exposure across multiple CED‐calculable alkylating agents provides minimal information beyond single‐agent assessment in reproductive‐age patients, whereas its incremental value was greater in pediatric patients.

**TABLE 1:** Additional patients identified as having at least intermediate‐risk exposure by summed total CED beyond single‐agent assessment.

Assessment	All patients, *n*	Pediatric patients, *n*	Reproductive‐age patients, *n*
Any single‐agent CED	730	124	606
Summed total CED	757	149	608
Additional patients identified	27	25	2
Relative increase, %	3.7	20.2	0.3

*Note:* The relative increase was calculated as the number of additional patients identified using the summed total CED divided by the number of patients identified using any single‐agent CED in each age group. Pediatric patients were defined as those aged < 15 years, and reproductive‐age patients as those aged 15–43 years. At least intermediate‐risk exposure was defined using age‐specific CED thresholds as described in the Methods section. In this table, the 124 and 149 pediatric patients correspond to 27.4% and 32.9% of the total exposed pediatric cohort (*n* = 453), respectively. The 606 and 608 reproductive‐age patients correspond to 13.7% and 13.7% of the total exposed reproductive‐age cohort (*n* = 4439), respectively.

Abbreviation: CED, cyclophosphamide equivalent dose.

**FIGURE 4:**
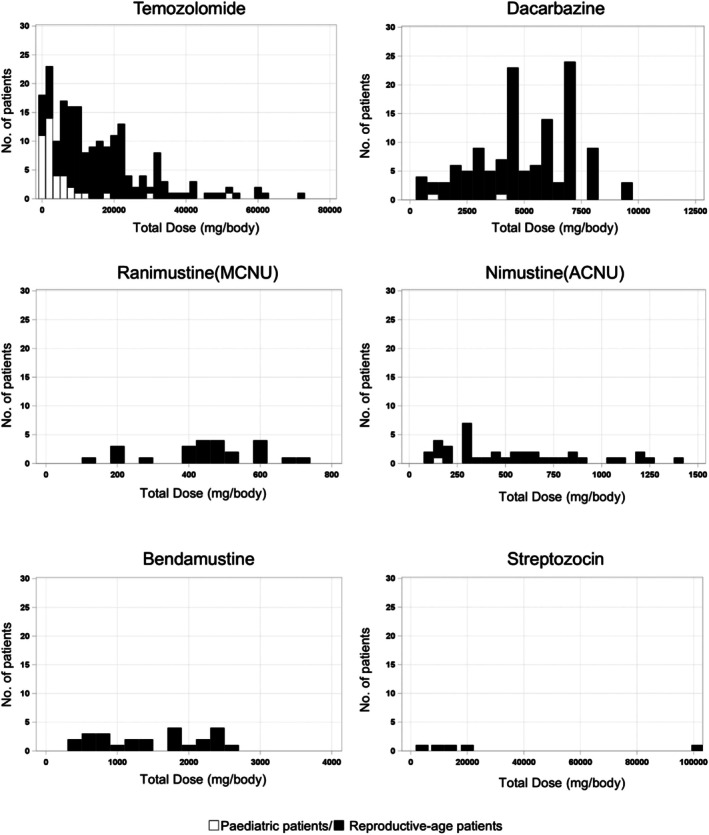
Distribution of cumulative prescribed doses for six alkylating agents not included in the current cyclophosphamide equivalent dose (CED) formula. Patients are stratified by age group as follows: Pediatric patients (< 15 years; open bars) and reproductive‐age patients (15–43 years; solid bars).

For alkylating agents not included in the current CED formula, the cumulative prescribed doses showed a wide distribution across patients (Figure 4). These agents were predominantly prescribed to reproductive‐age patients; furthermore, pediatric exposure was limited and, where present, tended to fall within relatively lower cumulative dose ranges. At the prescription‐record level, agents not included in the current CED formula accounted for 8.7% of all alkylating‐agent prescription records, approximately 90% of which were observed in reproductive‐age patients. Together with the prescription patterns shown in Figure 1, these findings suggest that part of the real‐world alkylating‐agent exposure, particularly in patients of reproductive age, was not incorporated into the total CED calculation.

## 4 Discussion

This claims‐based study described age‐specific alkylating‐agent exposure in pediatric and reproductive‐age females using the current CED framework. The principal finding was that the descriptive value and limitations of CED differed by age group. In pediatric patients, summing CED across multiple evaluable agents identified additional patients with at least intermediate‐risk exposure beyond single‐agent assessment. In reproductive‐age females, however, summed total CED added little beyond single‐agent assessment, whereas prescriptions for agents not included in the current CED formula were concentrated in this age group. These findings suggest that the current CED framework captures different aspects of real‐world alkylating‐agent exposure in pediatric and reproductive‐age females.

The single‐agent CED results also showed that prescription frequency alone does not adequately describe exposure burden. Cyclophosphamide was the most frequently prescribed alkylating agent in both age groups, but the proportion of cyclophosphamide‐exposed patients reaching at least intermediate‐risk exposure was lower than that for ifosfamide, melphalan, or busulfan. This pattern likely reflects differences in disease‐specific treatment regimens and cumulative dosing patterns across agents.

In pediatric patients, summed total CED identified additional patients with at least intermediate‐risk exposure compared with single‐agent assessment. Although this represented a small absolute increase within the exposed pediatric cohort, it supports the descriptive value of summed CED in pediatric patients. In contrast, in reproductive‐age females, summed total CED added little beyond single‐agent assessment. This suggests that the principal limitation in this age group is not multi‐agent integration among CED‐calculable agents but incomplete exposure capture for agents without established CED conversion coefficients.

The clustering of non‐CED‐agent prescriptions in reproductive‐age females may reflect both the historical development of the CED formula and age‐related differences in disease distribution. The original CED formula was developed from childhood cancer survivor cohorts [4] and has subsequently been referenced in fertility preservation guidance and risk‐stratification frameworks for adolescent and reproductive‐age patients [5, 6, 15]. In clinical practice, several agents not assigned CED conversion coefficients are used in disease contexts more commonly encountered in adolescent and adult populations. Examples include dacarbazine‐containing regimens for Hodgkin lymphoma [16, 17], bendamustine for indolent lymphomas [18, 19], temozolomide‐based treatment for adult glioblastoma [20, 21], and streptozocin for pancreatic neuroendocrine tumors [22]. These findings help clarify the descriptive scope and limitations of the current CED framework when applied across age groups and support further studies to determine whether and how agents outside the current formula should be considered within CED‐based or complementary exposure‐assessment frameworks.

This study has several limitations. First, ovarian reserve markers, premature ovarian insufficiency, and fertility outcomes were not assessed. Therefore, our findings should be interpreted as evidence regarding exposure capture rather than outcome prediction. Second, the database lacked information on patients' reproductive intentions and the clinical context of treatment decision‐making, including whether therapy was administered at initial diagnosis or for relapsed or refractory disease. Since observable follow‐up began at insurance enrolment, treatment received before cohort entry was not captured. Third, CED was estimated using age‐specific mean BSA values because individual body surface area data were unavailable. Fourth, claim‐based doses reflect billed treatment and may not perfectly correspond to administered doses. Finally, agents not included in the current CED formula were not incorporated into the total CED calculation, and the JMDC database primarily represents employee health insurance beneficiaries and their dependents in Japan, which may limit generalizability to other populations and healthcare systems. Nevertheless, a strength of this study is that it applied the CED framework as a descriptive exposure metric to population‐scale claims data across age groups and diverse disease contexts, describing both CED‐calculable exposure and exposure to agents outside the current formula.

In conclusion, this population‐scale claims‐based study showed that the current CED framework describes real‐world alkylating‐agent exposure differently by age group. Summed total CED provided additional exposure information mainly in pediatric patients, whereas in reproductive‐age females, a notable proportion of alkylating‐agent exposure involved agents without established CED conversion coefficients. Because reproductive outcomes were not assessed, these findings should be interpreted as evidence regarding exposure capture rather than outcome prediction. Further studies incorporating reproductive outcomes are needed to refine CED‐based exposure assessment and determine how agents outside the current formula should be considered in fertility preservation counseling.

## Funding

This study was supported by JSPS KAKENHI (Grant Number 24K12551).

## Ethics Statement

This study was conducted in accordance with the Declaration of Helsinki and the Japanese Ethical Guidelines for Medical and Biological Research Involving Human Subjects. The study protocol was approved by the Institutional Review Board of the Shiga University of Medical Science (R2026‐007).

## Consent

The requirement for informed consent was waived because of the use of de‐identified data.

## Conflicts of Interest

The authors declare no conflicts of interest.

## Supporting information


**Figure S1:** Distribution of cumulative prescribed dose for six alkylating agents included in the current cyclophosphamide equivalent dose (CED) formula. Distribution of the total cumulative prescribed doses (mg) for the six alkylating agents included in the CED formula evaluated in this study. Patients are stratified by age group as follows: pediatric (< 15 years; open bars) and reproductive‐age (15–43 years; solid bars) patients. The x‐ and y‐axis scales differ across panels to accommodate between‐drug differences in the cumulative prescribed doses and prescription frequencies, particularly for cyclophosphamide.

## Data Availability

The data used in this study are not publicly available because of the contractual restrictions imposed by JMDC Inc.
