# Supplementary figures and images for: Age‐Specific Gonadotoxic Alkylating‐Agent Exposure in Pediatric and Reproductive‐Age Females: A Claims‐Based Study Using Cyclophosphamide Equivalent Dose
Source: Reprod Med Biol. 2026 Aug 2;25(1):e70084. doi: 10.1002/rmb2.70084 (PMC13430070; doi:10.1002/rmb2.70084)

**Supplementary Figure S1**


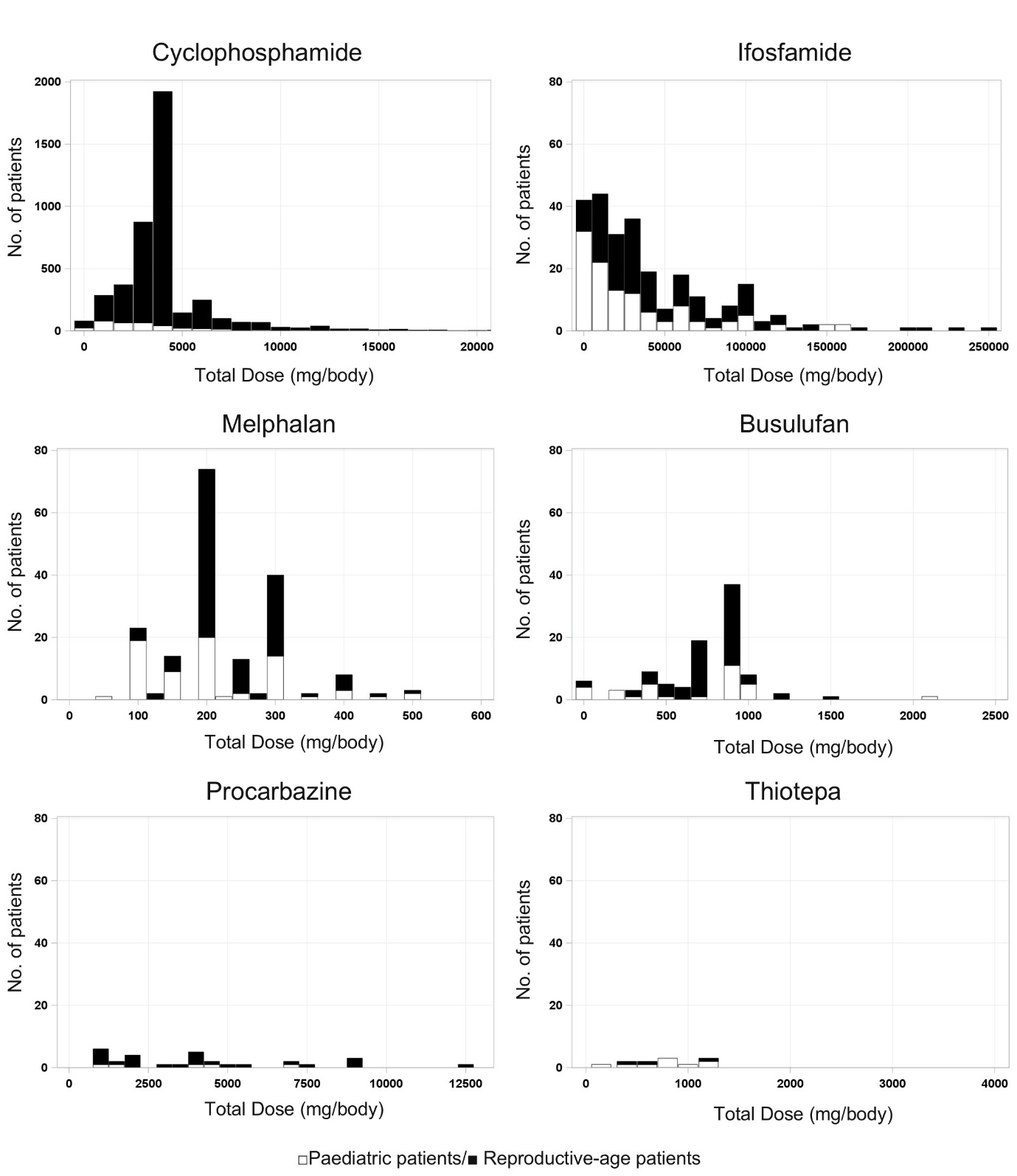

Supplement: Supplementary file 1 — Figure S1: Distribution of cumulative prescribed dose for six alkylating agents included in the current cyclophosphamide equivalent dose (CED) formula. Distribution of the total cumulative prescribed doses (mg) for the six alkylating agents included in the CED formula evaluated in this study. Patients are stratified by age group as follows: pediatric (< 15 years; open bars) and reproductive‐age (15–43 years; solid bars) patients. The x‐ and y‐axis scales differ across panels to accommodate between‐drug differences in the cumulative prescribed doses and prescription frequencies, particularly for cyclophosphamide. [file RMB2-25-e70084-s001.docx]
